# miR-203 Inhibits Alcohol-Induced Hepatic Steatosis by Targeting Lipin1

**DOI:** 10.3389/fphar.2018.00275

**Published:** 2018-04-04

**Authors:** Xiao-Yu Cheng, Jun-Da Liu, Xin-Yi Lu, Xing Yan, Cheng Huang, Xiao-Ming Meng, Jun Li

**Affiliations:** The Key Laboratory of Major Autoimmune Diseases, The Key Laboratory of Anti-inflammatory and Immune Medicines, Ministry of Education, Anhui Institute of Innovative Drugs, Institute for Liver Diseases, School of Pharmacy, Anhui Medical University, Hefei, China

**Keywords:** alcoholic fatty liver, miR-203, Gao-binge, Lipin1, lipid metabolism

## Abstract

Alcoholic liver disease (ALD) is a global liver disease which characterized by liver inflammation, fatty liver, alcoholic hepatitis, or liver cirrhosis. Alcohol abuse is one of the main reasons for liver disease. Alcoholic fatty liver (AFL) disease is the early stage of ALD and associated with the excessive lipids accumulation in hepatocytes as well as oxidative stress. MicroRNA-203 (miR-203) is known to suppress the proliferation and metastasis of hepatocellular carcinoma, but the role in the progression of alcoholic liver disease is not clear and is warranted for further investigation. In the present study, we have found the expression of miR-203 is down-regulated in Gao-Binge alcoholic mice model and ethanol-induced AML-12 cell lines *in vitro*. Furthermore, over-expression of miR-203 decrease the lipids accumulation in liver and ethanol-induced AML-12 cells. Mechanistically, we identified that Lipin1 is a key regulator of hepatic lipid metabolism, and acts as a downstream target for miR-203. In summary, our results suggested that over-expression of miR-203 inhibited the liver lipids accumulation and the progression of AFL by targeting Lipin1.

## Introduction

Alcoholic liver disease (ALD) is one of the most common liver diseases in the world and it becomes a major cause of affecting the morbidity and mortality worldwide (Ramaiah et al., 2004). In Asia, alcohol consumption also a main cause of chronic liver disease because people have an increase alcohol consumption in recent years (Kwon et al., 2014). Excessive alcohol consumption can develop alcoholic hepatitis, fibrosis, and cirrhosis (Szabo et al., 2012). Metabolism of alcohol triggers activation of the innate immune cells in the liver, increases the generation of cellular NADH. Aldehyde dehydrogenases is an important biological enzyme involved in alcohol metabolism. Metabolism of alcohol can impair oxidation and tricarboxylic acid cycle activity. This leads to a lot of free fatty acid overload in turn, increased synthesis and secretion of triacylglycerol (TG), very low-density lipoprotein (VLDL), which contributes to the development and pathogenesis of ALD. Although fatty liver in the past was thought to be a benign stage, more and more studies show that the liver can more susceptible to injury by other agents if fat accumulation. It has been said that patients who consume alcohol chronically develop steatosis due in part to alterations in lipid metabolism (Crabb and Liangpunsakul, 2006) and without treatment may develop to advanced stages of ALD that include liver fibrosis and liver cirrhosis (MacSween and Burt, 1986).

The miRNAs are small non-coding RNAs, which control gene expression by targeting mRNAs and triggering either translational repression or RNA degradation (Tang et al., 2008). There is an abundance of evidences reports that several microRNAs (miRs) contribute to ALD (Chen et al., 2013; McDaniel et al., 2014). MiR-203 has been found be a tumor suppressor gene, which can inhibit the development of tumor by regulating cell growth, proliferation, and metastasis (Takeshita et al., 2012). MiR-203 can suppress the hepatocellular carcinoma cells proliferation by targeting HOXD3 and the progression is conducted through EGFR signaling pathway (Wang et al., 2016). MiR-203 can also suppress the hepatocellular carcinoma by targeting oncogene ADAM9 and oncogenic long non-coding RNA HULC (Wan et al., 2016). In addition, miR-203 can regulate liver fibrotic and TGF-β induced proliferation of hepatic stellate cells by targeting TRPV4 (Song et al., 2014). However, the functional significance of miR-203 in ethanol-induced hepatic steatosis has not been studied.

The protein encoded by the Lpin1 gene (Lipin1) is mammalian Mg^2+^-dependent phosphatidate phosphatase type (PAP), considerable studies have been implicated the regulate role of cellular lipid metabolism in a variety of tissues, including liver (Finck et al., 2006). Peterfy and his groups are first discovered Lipin1 by using a positional cloning approach to identify the genetic mutation responsible for the fatty liver dystrophic (FLD) mouse phenotype (Peterfy et al., 2001). The dual seemingly paradoxical functions of Lipin1 for one hand as a PAP enzyme required for lipid synthesis and that can also promote fatty acid oxidation as a transcriptional coactivator (Chen et al., 2015). Peroxisome proliferator-activated receptor-α (PPAR-α) plays a key role in the pathogenesis of ALD (Fischer et al., 2003). Lipin1 localizes to the nucleus and complex with PPAR-α/PGC-1α in a form, which stimulates fatty acid oxidation in the liver. On the other hand, Lipin1 subcellular localization regulates sterol response element binding protein-1 (SREBP-1) signaling and involves in the biosynthesis of TG (Hu et al., 2012).

The aim of the present study was to investigate the role of miR-203 in alcoholic liver disease and to further elaborate the possible molecular mechanisms involved in miR-203 function in alcoholic liver disease. We examined the miR-203 expression in liver tissue from EtOH-fed mice, control diet-fed mice and AML-12 cell lines. Meanwhile, we packaged lentivirus to over-expressed miR-203 in the liver by tail vein injection with C57BL/6J mice *in vivo*, and transfected miR-203 mimics into AML-12 cell lines to over-expressed miR-203 *in vitro*. Then the impacts of miR-203 on lipid metabolism in liver and AML-12 cell lines are examined. Furthermore, we proved that miR-203 could regulate the biological progression of ALD via directly targeting Lipin1 expression. The subcellular localization of Lipin1 is linked with miR-203, over-expressed miR-203 can inhibited cytoplasmic localization of Lipin1 in mouse AML-12 cells. These results might suggest the functions of miR-203 and its roles in ALD progression, which may provide a scientific and novel therapeutic strategy for alcoholic fatty liver (AFL).

## Materials and Methods

### Animal, Mouse Model of ALD

All 8-week-old male C57BL/6J mice were provided by the Experimental Animal Center of Anhui Province used for ALD model. The animal experimental procedures approved by the Ethics Committees of Anhui Medical University, and were reviewed and performed in accordance with the Guideline of Animal Care and Use Committee of Anhui Medical University. For all experiments, mice were divided randomly into control diet (CD)-fed group and EtOH-fed group. ALD model was constructed by reference the National Institute on Alcohol Abuse and Alcoholism (NIAAA), more details can be found in the article (Wu et al., 2016). Modeling process includes a liquid diet adaptation period (5 days), modeling (10 days), gavage (1 time) and specimens (1 day), a total of 16 days. The EtOH-fed mice were fed ethanol (5% v/v) liquid diets (LD) for 10 days plus a single binge ethanol administration (5 g/kg, body weight, 20% ethanol) by gavage, and the CD-fed mice were fed and gavaged with isocaloric maltose-dextrin. All diets were prepared fresh daily. Mice were anesthetized after the last gavage alcohol injection, and blood and liver tissues were collected for the further analysis. Plasma was stored at -80°C. Portions of liver tissue were frozen immediately in liquid nitrogen, whereas others were fixed in 10% neutral-buffered formalin for hematoxylin and eosin (H&E) staining and Oil red O staining, or embedded in frozen specimen medium (TissueTek OCT compound; Sakura Finetek, Torrance, CA, United States).

### Lentivirus Vector Administration

A 200-bp DNA fragment corresponding to pre-Mir-203 and its flanking sequences was amplified from mouse genomic DNA and was subsequently cloned into lentiviral vector (GenePharma, Shanghai). The packaged lentiviruses were named Lenti-miR-203. The empty (untransformed) lentiviral vector named Lenti-NC served as control. Then Lenti-miR-203, Lenti-NC were administered at a dose of 2 × 10^8^ transducing units per animal by tail vein injection (200 μl total volume) using a 30 gauge ultra-fine insulin syringe before the ALD model construction.

### Cell Culture

AML-12 cells were purchased from the Type Culture Collection of the Chinese Academy of Sciences (Shanghai, China) and cultured in F12 medium (Hyclone, United States) supplemented with 10% fetal bovine serum (Clark). All cells were incubated at 37°C in a humidified incubator containing 5% CO_2_.

### miRNA Mimics, Inhibitor, and siRNA Transfection

To over-expressed and down-regulated the expression of miR-203, AML-12 were transfected with miR-203 mimics or inhibitor by using lipofectamine 2000 (Invitrogen, United States) according to the manufacturer’s instructions. MiR-203 mimics and control mimics, miR-203 inhibitor and control inhibitor were synthesized by Biomics biotech (Jiangsu, China). In order to knock down Lipin1, Lipin1 small interfering (si) RNA or negative control RNA (GenePharma, Shanghai) using lipofectamine 2000 transfected in AML-12 cell. After 6 h transfection, the medium was changed to F12 and ethanol was added. Cells were maintained at 37°C in a CO_2_ incubator for 24 h and then collected for Western blot analysis or other experiments. The RNA oligo sequences were as follows: Negative control: sense 5′-UUCUCC GAACGUGUCACGU TT-3′, antisense 5′-ACGUGACACGUUCGGAGAATT-3′, Lipin1-siRNA: 5′-GGUUGACGCCAAAGAAUAATT-3′, 5′-UU AUUCUUUGGCGUCAACCTT-3′.

### RNA Isolation and Quantitative Real-Time PCR

Total RNA, including miRNA was extracted from mouse liver tissues, AML-12 cells using TRIzol reagents (Invitrogen, United States), and the first-strand cDNA was synthesized using transcriptor first-strand cDNA synthesis kit (TaKaRa, Shiga, Japan) according to the manufacturer’s instructions. For miR-203, the procedure was performed according to the manuscript of the one-step miRNA qRT-PCR Detection Kit (Biomics, Nantong, Jiangsu, China). Real-time quantitative PCR analyses for mRNA of Lipin1, PPAR-α, SREBP-1, IL-6, TNF-α, and GAPDH were performed in a detection system with SYBR-Green Master Mix (TaKaRa, Shiga, Japan). qRT-PCR primers were purchased from Invitrogen. The mRNA level of GAPDH was used as an internal control. Primer sequences were listed as following: Lipin1 (forward 5′-GCCAGGTGTTTGTGACGGTGA-3′; reverse 5′-GCTGGCTTTCCATTCTCGCA-3′), PPAR-α (forward 5′-TAACCCGCCTTTCGTCATAC-3′; reverse 5′-TGGC AGCAGTGGAAGATG-3′), SREBP-1 (forward 5′-ACACA GCAACCAGAAACTCAAG-3′; reverse 5′-AGTGTGTCCTCCA CCTCAGTCT-3′), IL-6 (forward 5′-GA GGATACCACTCC CAACAGACC-3′; reverse 5′-AAGTGCATCATCGTTGTTCA TACA-3′), TNF-α (forward 5′-CATCTTCTCAAAATTCGAGT GACAA-3′; reverse 5′-TGGGAGTAGACAAGGTACAACCC-3′), GAPDH (forward 5′-CCAACCGCGAGAAGATGA-3′; reverse 5′-CCAGAGGCGTACAGGGATAG-3′). All experiments were performed in triplicate and repeated at least three times.

### Protein Isolation and Western Blot Analysis

Protein was extracted from AML-12 cells or liver tissues using RIPA lysis buffer (Beyotime, China) supplemented with 1% PMSF. After centrifugation for 30 min at 4°C at 12000 rpm, upper supernatant was collected. Protein was separated on 8–12% SDS-polyacrylamide mini-gels, then transferred onto polyvinylidene fluoride membranes (Millipore, Billerica, MA, United States). After blocked in 5% non-fat milk for 3 h, membranes were washed in TBS-Tween20 buffer three times per 10 min, and incubated with specific primary antibodies overnight. Primary antibodies Lipin1 (ab181389), PPAR-α (ab8934), SREBP-1 (ab28481), FASN (ab128856) were purchased from Abcam. Primary antibodies IL-6 (BS6419) was purchased from Bioworld. β-Actin (bs-0061R) and TNF-α (bs-10802R) were purchased from Bioss. Histone3 (17168-1-AP) was purchased from Proteintech. Then the membranes were washed for three times with TBS-Tween20 buffer, following incubation with the corresponding horseradish peroxidase conjugated secondary antibody for 1 h and then developed onto chemiluminescence. Western blotting detection system using ECL-chemiluminescent kit (ECL-plus, Thermo Scientific). Quantitative densitometric analyses of immunoblotting images were performed using ImageJ software. The experiment was repeated for three times.

### Histopathology

The liver tissues were fixed with 4% paraformaldehyde for 24 h and embedded in paraffin blocks and stained for routine histology. H&E staining and Oil red staining were performed according to a standard procedure. The pathological changes were assessed and photographed under an Olympus microscope.

### Immunohistochemistry

The liver tissue was fixed in 10% neutral buffered formalin solution and embedded in paraffin for routine histology. Slides were de-paraffinized in xylene and dehydrated in alcohol, and antigen retrieval was achieved by microwaving in citric saline for 15 min. Then sections were covered with 3% H_2_O_2_ for 10 min to block endogenous peroxidase activity. After being blocked with 5% bovine serum albumin, the slides were incubated with primary antibody against Lipin1 overnight at 4°C. After rinsing, the sections were incubated with corresponding secondary antibody for 1 h at room temperature. Antigenic sites were visualized 3,3′-diaminobenzidine tetrahydrochloride (DAB) staining. The slides were counterstained with hematoxylin for 1 min, dehydrated and then observed using an Olympus microscope.

### Immunofluorescence

Cells were fixed with 4% paraformaldehyde in phosphate-buffered saline and permeabilized with 0.2% Triton X-100 in phosphate-buffered saline. Then cells were washed twice with PBS and blocked with 1% BSA and 10% fetal bovine serum in PBS for 20 min. Fixed cells were incubated with 1:2000 fluorescein isothiocyanate-conjugated phalloidin (Sigma, St. Louis, MO, United States) or antibodies as indicated. Cells were counterstained with 4, 6-diamidino-2-phenylindole (DAPI) (Calbiochem, San Diego, CA, United States) and imaged with Olympus microscope.

### TG and TCH Analysis and ALT/AST Activity Assay

Alanine aminotransferases (ALT) assay kit, aspartate aminotransferases (AST) assay kit, triglyceride (TG) and total cholesterol (TCH) assay kits were from Jiancheng Biology Institution PeproTech (Nanjing, Jiangsu, China). The serum or liver tissue levels of TG and TCH in mice were assayed using TG and TCH assay kits according to the protocols. ALT and AST levels in serum from C57BL/6J mice with ALD were analyzed by using ALT and AST activity assay kits according to the protocols recommended by the manufacturer. The absorbance was measured at 510 nm.

### Plasmid Transfection and Luciferase Reporter Assays

In order to validate the LPIN1 gene was a target of miR-203, we packaged a reporter plasmid containing luciferase with the 3′ UTR sequence of Lipin1 mRNA. The specificity of miR-203 targeting Lipin1 mRNA was ascertained by co-transfection of pSicoR/miR-203 and pMIR-Lipin1-wild, pMIR-Lipin1-mut or the control vectors into AML-12 cells using Lip2000, and the results were determined by the relative activity of firefly luciferase unit (RLU) at 48 h post-transfection using a dual luciferase reporter assay kit (Promega, Madison, WI, United States) followed the manufacturer’s instructions.

### Nuclei and Cytoplasmic Protein Extraction

Nuclei and cytoplasmic protein were extracted from cells using special lysis buffer (BB-36021, BestBio) followed the manufacturer’s instructions.

### Statistical Analysis

All the experiments were performed at least three times independently, and typical results are shown with values expressed as means ± SD. An analysis of variance (ANOVA) and Student’s *t*-test were used to estimate the differences among groups. A *p*-value < 0.05 was considered statistically significant. All statistical analyses were performed with Prism software program for Windows (Graph Pad Software).

## Results

### miR-203 Was Down-Regulated in EtOH-Fed Mice and EtOH-Induced AML-12 Cells

**FIGURE 1:**
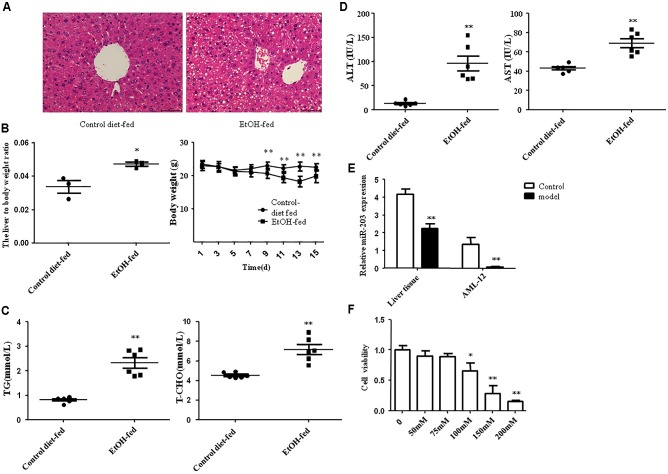
miR-203 was down-regulated in EtOH-fed mice and EtOH-induced AML-12 cells. **(A)** Representative hematoxylin and eosin (H&E) staining of liver tissues (×400). **(B)** Body weights and the liver to body weight ratio after ethanol feeding. **(C)** Hepatic triglyceride (TG) and total cholesterol (TCH) levels. **(D)** Serum ALT and AST levels. **(E)** One-step qRT-PCR for miR-203 expression in EtOH-fed mice liver tissues compared to normal liver tissues and AML-12 cell line. **(F)** AML-12 cell line was treated with ethanol (0, 50, 75, 100, 150, 200) mM for 24 h the cell viability. ^∗^*p* < 0.05, ^∗∗^*p* < 0.01 versus control group.

All male C57BL/6J mice with chronic alcohol feeding were characterized by immune cell activation, inflammation, injury, and steatosis in the liver. In order to evaluate the expression level of miR-203 during ALD in mouse EtOH-fed models, H&E staining was used to evaluate the degree of liver injury. Histopathological analysis showed that the liver tissues in EtOH-fed mice exhibited fat vacuoles, liver cell cord derangement, intercellular spaces dilatation and inflammatory cell infiltration, while CD-fed mice showed normal lobular architecture with central veins and radiating hepatic cords (**Figure 1A**). Furthermore, the body weights of both EtOH-fed mice and CD-fed mice were gradually decreased at the first phase days, and then increased slightly after the adaptive phase. However, the body weights of EtOH-fed mice were significantly lower than those of the control group, and the liver to body weight ratio in the EtOH group was remarkably higher than that in the control group at the end of model building (**Figure 1B**). To determine the effect of EtOH ingestion on lipid homeostasis, we collected the mice serum to detect the TG, TCH, ALT, AST. As shown in **Figure 1C**, there was a dramatic increase in serum TG and TCH levels in EtOH-fed mice. Moreover, these metabolic changes were associated with a significant increase in serum levels of ALT and AST (**Figure 1D**), suggesting that EtOH consumption induced hepatocellular injury in this mouse model. In according with the above data, the number of lipid droplet was significantly increased in liver tissue of EtOH-fed mice compared to the CD-fed mice.

Next, one-step real-time PCR assay showed that miR-203 was reduced as compared with normal liver tissues (**Figure 1E**). Furthermore, we treated the AML-12 cells with different concentration of EtOH for 24 h. We found that the cell viability was decreased in dose-dependent manner (**Figure 1F**). Then we stimulated AML-12 cells with 100 mM EtOH for 24 h to detect the expression of miR-203, the result was the same as liver tissues.

### miR-203 Inhibited Liver Lipids Accumulation *in Vivo*

**FIGURE 2:**
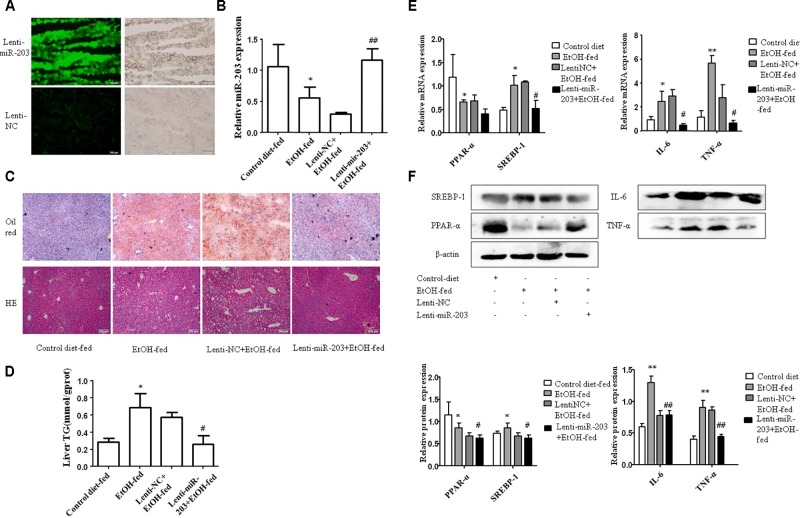
miR-203 inhibited liver lipids accumulation *in vivo*. **(A)** Lenti-mir-203 and Lenti-NC expression in mice liver. **(B)** Expression of miR-203 was confirmed by qRT-PCR. **(C)** The liver tissues H&E and Oil Red O staining (x100). **(D)** Liver triglyceride (TG) levels. **(E,F)** qRT-PCR and Western blot analysis for mRNA and protein expression of lipid metabolism markers PPAR-α^∗^ SREBP-1, inflammation markers IL-6, TNF-α. ^∗^*p* < 0.05, ^∗∗^*p* < 0.01 versus control group. ^#^*p* < 0.05, ^##^*p* < 0.01 versus NC group.

To explore the regulatory effect of miR-203 on Gao-binge alcoholic mice liver lipids accumulation, 8-week-old male C57BL/6J mice were tail vein injected with Lenti-miR-203 or Lenti-NC before the model construction. Lentivirus vector have fluorescence, so the miR-203 over-expression can be analysis by microscope (**Figure 2A**). qRT-PCR showed that liver miR-203 expression was significantly increase after treated with Lenti-miR-203 (**Figure 2B**). The liver H&E and Oil Red O staining have shown hepatic lipid accumulation levels were significantly higher than controls in both chronic alcohol diet-fed mice and Lenti-NC infected mice. However, administration of Lenti-miR-203 greatly reduced the development of AFL (**Figure 2C**). Hepatic triglyceride assay also showed that liver triglyceride levels were markedly higher in chronic alcohol diet-fed mice and Lenti-NC infected mice (**Figure 2D**). Additionally, Western blotting further revealed that lentivirus-mediated ectopic miR-203 expression resulted in significant reduction of lipid metabolism markers including SREBP-1 and inflammation markers IL-6, TNF-α (**Figures 2E,F**).

### miR-203 Inhibited Hepatocyte Lipids Accumulation *in Vitro*

**FIGURE 3:**
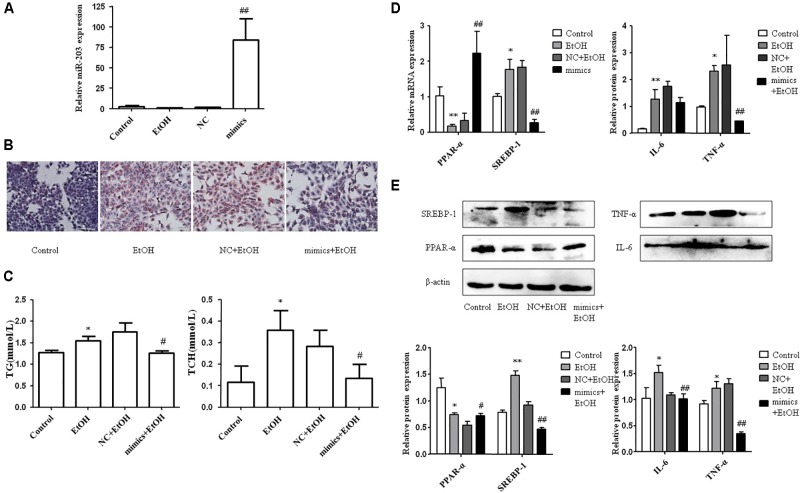
miR-203 inhibited hepatocyte lipids accumulation *in vitro*. **(A)** Transfection effect of miR-203 mimics was confirmed by qRT-PCR. **(B)** The cellular Oil Red staining (x200). **(C)** The cellular TG and TCH levels. **(D,E)** qRT-PCR and “Western blot” analysis for mRNA and protein expression of lipid metabolism markers PPAR-α^∗^ SREBP-1, inflammation markers IL-6, TNP-α. ^∗^*p* < 0.05, ^∗∗^*p* < 0.01 versus control group. ^#^*p* < 0.05, ^##^*p* < 0.01 versus NC group.

To explore the effect of miR-203 on hepatocyte lipids accumulation *in vitro*, AML-12 cells were transiently transfected with miR-203 mimics, miR-203 inhibitor, or miR controls. qRT-PCR showed that miR-203 expression was significantly increased after miR-203 mimics transfection (**Figure 3A**). The cellular Oil Red staining indicated over-expression of miR-203 significantly suppressed the cellular lipids accumulation (**Figures 3B,C**). Further experiment showed that over-expression of miR-203 in AML-12 cells could promote the protein and mRNA level of PPAR-α and inhibit the level of SREBP-1, IL-6, or TNF-α (**Figures 3D,E**).

### miR-203 Directly Regulated the Expression of LPIN1

**FIGURE 4:**
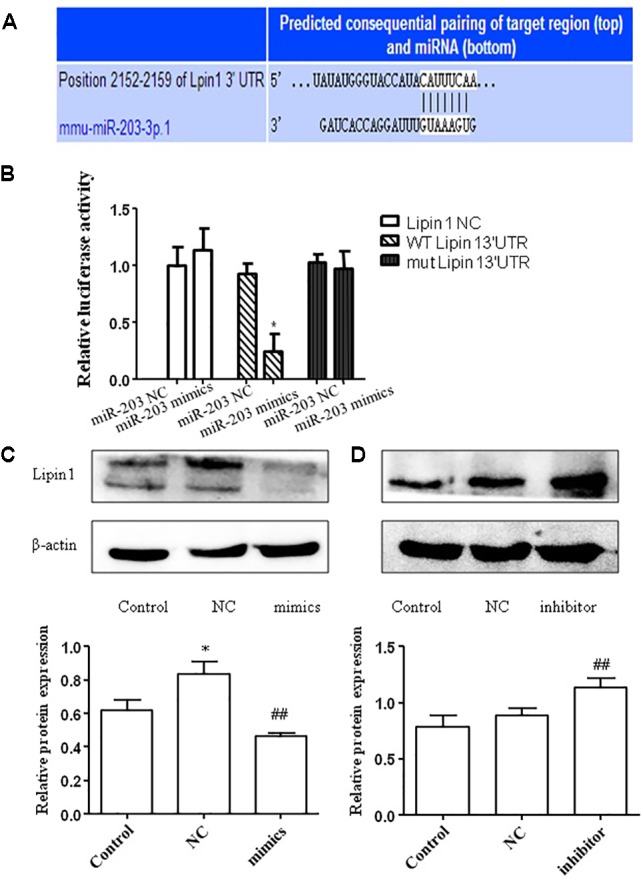
miR-203 directly regulated the expression of Lipin1. **(A)** Bioinformatics analyses show the seed sequence of miR-203 bind to the 3′-UTR of LPIN1 mRNA. **(B)** “Wild type” 3′-UTR of LPIN1 gene was cloned into the firefly and Renilla reporter plasmid. The LPIN1-3′ UTR constructs or blank plasmid were transfected into AML-12 cells with control or miR-203 mimics, followed by dual luciferase assays. **(C,D)** Western blot analysis for protein expression of LPIN1 after transfected with miR-203 mimics or miR-NC, mir-203 inhibitor or inhibitor-NC. ^∗^*p* < 0.05, ^∗∗^*p* < 0.01 versus control group. ^#^*p* < 0.05, ^##^*p* < 0.01 versus NC group.

We then used miRBase and TargetScan to search for the target genes of miR-203. Interestingly, we found that the 3′-UTR of LPIN1 had putative binding sites with miR-203 (**Figure 4A**). To further test whether Lipin1 is a target of miR-203, the 3′-UTR was cloned into a luciferase expression vector to evaluate its response to miR-203. Co-transfected the luciferase reporter with the miR-203 mimics into AML-12 cells showed the 3′-UTR conveyed decreased expression (**Figure 4B**). In our experiments, we found that the mRNA expression of Lipin1 could be decreased by miR-203 mimics (**Figure 4C**), and the Lipin1 mRNA level was increased in cells after transfected with miR-203 inhibitor (**Figure 4D**). Western blotting further revealed that the protein expression of Lipin1 was also inversely correlated with miR-203 (**Figure 4**). Taken together, our results suggested that Lipin1 was a target of miR-203 in AML-12 cells.

### Knocked Down Lipin1 Contributed to Inhibit Hepatocyte Lipids Accumulation *in Vitro*

**FIGURE 5:**
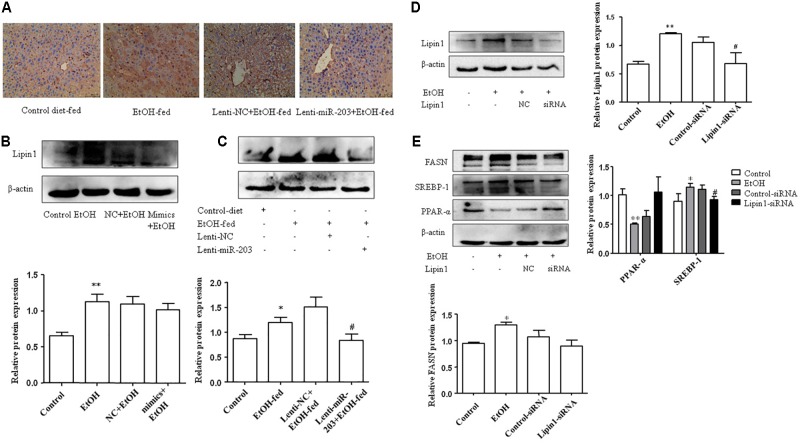
Knocked down Lipinl contributed to inhibit hepatocyte lipids accumulation *in vitro*. **(A)** IHC analysis on liver tissues between the four groups. **(B)** Western blot analysis for Lipinl expression on mice liver tissues. **(C)** AML-12 cells were transfected with miR-203 mimic or miR-NC and then stimulated with ethanol 100 mM 24 h, Western analysis for Lipinl protein expression. **(D)** AML-12 cells were transfected with Lipinl-siRNA or control siRNA and then stimulated with ethanol 100 mM 24 h, Western analysis for Lipinl protein expression. **(E)** Western analysis for PPAR-α, SREBP-1, FASN protein expression after transfection. ^∗^*p* < 0.05, ^∗∗^*p* < 0.01 versus control group. ^#^*p* < 0.05, ^##^*p* < 0.01 versus NC group.

To identify the changes in the Lipin1 expression between EtOH-fed mice and CD-fed mice, IHC analysis and Western blot on liver tissues between the four groups were performed. Lipin1 expression was negligible in liver tissue from CD-fed mice and Lenti-NC mice but highly expressed in liver tissue from EtOH-fed mice and Lenti-miR-203 mice (**Figures 5A,B**). In AML-12 cells, ethanol increased total Lipin1 protein levels, however, transfected with miR-203 mimics the expression of Lipin1 was increased (**Figure 5C**). In order to explored the effect of Lipin1 on hepatocyte lipids metabolism, we knocked down Lipin1 by transfecting Lipin1-siRNA in AML-12 cells. Western blot showed that the expression of Lipin1 was down-regulated after transfection (**Figure 5D**). Further experiment showed that knocked down of Lipin1in AML-12 cells could promote the protein of PPAR-α, but inhibit the expression of SREBP-1 and FASN (**Figure 5E**).

### miR-203 Inhibited Cytoplasmic Localization of Lipin1 in Mouse AML-12 Cells

**FIGURE 6:**
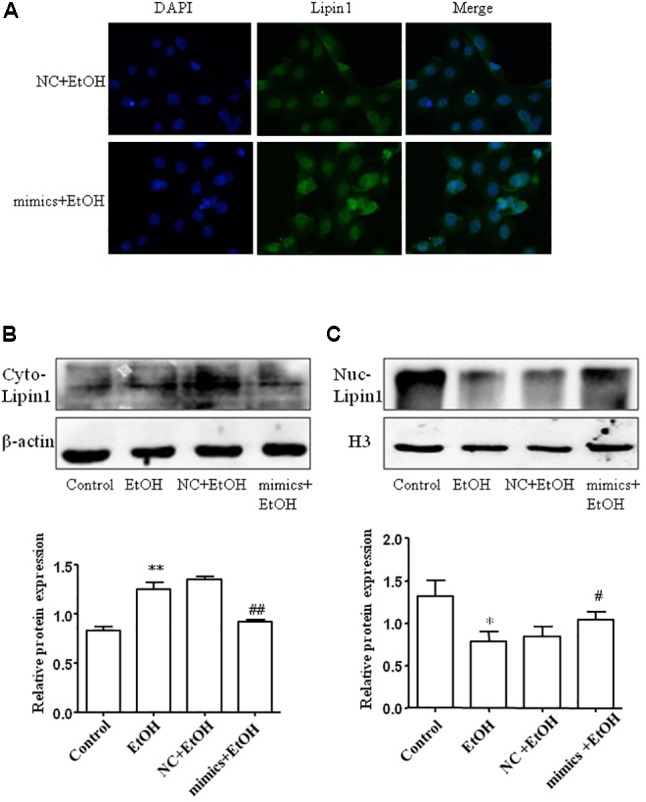
miE-203 inhibited cytoplasmic localization of Lipin1 in mouse AML-12 cells. **(A)** Representative photomicrographs of immunofluorescence Lipin1 (green) or DAPI (blue) m AML-12 cells transfected with miR-203 mimics or NC and then treated with ethanol (100 mM) for 24 h (x200). **(B,C)** Representative western blotting analysis of the Lipin1 protein expression levels in cytoplasm (Cyto) or nucleus (Nuc).

To assess the subcellular localization of Lipin1 in response to miR-203 regulatory in AML-12 cells. Immunofluorescent staining of nuclei and Lipin1 confirmed that endogenous Lipin1 was present predominantly in the cytoplasm. Furthermore, treatment with ethanol dramatically increased the intensity of Lipin1 staining in the cytoplasm, as compared to controls, suggesting an increase of Lipin1 protein expression, but over-expressed miR-203 the Lipin1 cytoplasmic accumulation was reduced and nuclei accumulation was increased (**Figure 6A**). Then we assessed the subcellular localization of Lipin1 in response to miR-203 regulatory in AML-12 cells. AML-12 cells were transiently transfected with miR-203 mimics and then cultured in the presence of ethanol, and extracts of these cells were fractionated into cytosol and nuclei, followed by Western blotting analysis. The increase in endogenous Lipin1 protein induced by ethanol was observed strictly in the cytosolic fractions, however, the expression of Lipin1 was decreased when over-expression of miR-203 (**Figure 6B**). Conversely, these results were contrary to the nuclei fractions (**Figure 6C**).

## Discussion

The most common approach to treatment with ALD patients is eliminated alcohol intake because of continued alcohol ingestion is the single most important risk factor for the disease progression (Gao and Bataller, 2011). Increased lipogenesis and excessive lipid accumulation can directly damage hepatocytes after chronic alcohol exposure, and then followed with an inflammatory response, oxidative stress and cytokine production, resulting in the development of AFL disease (Tessari et al., 2009). Therefore, inhibiting hepatic lipid accumulation effectively during alcohol exposure can prevent further liver damage. MicroRNAs have been recently identified as master regulators to control the tolerance of ethanol addiction (Miranda et al., 2010). MiR-203 as a tumor suppress gene, studies have found hepatic over-expression of miR-203 could facilitate the initiation of the liver by targeting SOCS3 through IL-6/STAT3 signaling pathway (Chen et al., 2017). Microarray and qRT-PCR has been utilized to detect serum miRNAs pattern in a rat ASH model, they found miR-203 was down-regulated in serum and liver (Chen et al., 2013). To the best of our knowledge, there are no data about the role of miR-203 in AFL. In this study, we found miR-203 levels in liver tissue from EtOH-fed mice were significantly lower than those in CD-fed mice. To elucidate the potential function of miR-203 in the progression of ALD pathology, we activated AML-12 cells with 100 mM EtOH *in vitro*. The data showed that miR-203 was significantly down-regulated after stimulation. Furthermore, we over-expressed miR-203 by injecting lentivirus encoding miR-203 mimics through the right caudal vein in mice before ALD model construction. Both H&E and Oil Red O staining pathological staining results showed that miR-203 reduced alcoholic liver steatosis. Then we verified the *in vivo* findings *in vitro*, further demonstrated that miR-203 suppressed hepatic lipid accumulation.

SREBP-1 is an important transcription factor, which accelerates fatty acid synthesis. PPAR-α is a nuclear hormone receptor can modulate transport and oxidation of fatty acids. These two genes could modulate the lipogenesis and β-oxidation in hepatocytes (Lu et al., 2015). SREBP-1 could directly regulate the transcriptions of genes involved in synthesis and uptake of TG, TC, and fatty acids, such as FAS (Horton et al., 2002). SREBP-1c has been implicated in the pathogenesis of AFLD, which can be activated by ER stress, LPS and TNF-α, both of these are increased in alcoholic liver disease (Sozio et al., 2010). PPAR-α acts as a heterodimer with retinoid X receptor (RXR), studies have indicated that ethanol consumption impaired activation of PPAR-α and contributed to the development of AFL, and possibly inflammation (Crabb and Liangpunsakul, 2006). Our data demonstrated that SREBP-1 expression was up-regulated but PPAR-α was decreased in response to alcohol exposure, and over-expression of miR-203 suppressed the ethanol-mediated induction of TG and TCH. In brief, all these findings suggest that up-regulation of miR-203 inhibited the fat accumulation, suggesting that miR-203 may be involved in the development of hepatic steatosis.

Inflammation is a critical driver of hepatic disease processes in ALD (Louvet and Mathurin, 2015). High-fat diet-fed mouse model has found produced inflammatory and fibrotic changes associated with tissue remodeling (Zhang et al., 2017). Besides, we further evaluated the effect of ethanol on the expression of inflammation cytokines, such as TNF-α, IL-6. The results had shown alcohol feeding induces TNF-α, IL-6, but miR-203 reduce the inflammation reaction. Combined with the above observations, we realized that alcoholic liver injury mainly resulted from disordered lipid metabolism and that miR-203 could inhibit the inordinate metabolism and inflammation to inhibit alcoholic steatosis and injury.

Lipin1 is a member of Lipin family, which contains Lipin2 and Lipin3. Today most study focus on Lipin1 and there is little known about Lipin2 and Lipin3. Lipin1, a bi-functional protein, regulates fatty acid utilization and triglyceride biosynthesis (Kim et al., 2013; Kajimoto et al., 2016). These two functions of Lipin1 are necessary for full of the biological activity (Ren et al., 2010). Lipin1 around nuclear might be needed to interact with PPAR-α and PPAR-γ to regulate fatty acid oxidation. And delipidation of nuclear Lipin1 may impair the complex, which will lead to decrease in fatty acid oxidation (Zeng et al., 2017). Down-regulation of hepatic Lipin1 expression decreases the TG level in the liver and blood circulation (Kajimoto et al., 2016). However, little is known about the role of Lipin1 in AFL. We investigated that Lipin1 was significantly up-regulated in mice alcoholic fat liver tissues as well as ethanol treated AML-12 cells. Then luciferase assays have found the links with miR-203 and Lipin1. Besides, our study found that protein expression of Lipin1 in AML-12 cells were negatively regulated by alteration of miR-203. It suggests that miR-203 plays an important role in AFL partly by directly targeting Lipin1. Then we knocked down Lipin1 by transfection with Lipin1-siRNA, the protein of PPAR-α was increased compared with control-siRNA. However, SREBP-1 and FASN protein expression was decreased.

As previously mentioned, Lipin1, determines whether fatty acids are incorporated into triglycerides or undergo mitochondrial β-oxidation. In the cytoplasm, Lipin1 as a Mg^2+^-dependent PAP causes triglycerides accumulation and promotes phospholipids synthesis by PAP1 activity in the cytosol (Ishimoto et al., 2009). In the nucleus, Lipin1 acts as a transcriptional co-activator and has been linked to fatty acid oxidation (Barroso et al., 2011). Our studies demonstrated ethanol promoted cytoplasmic localization of Lipin1, but over-expression miR-203 the LPIN1 gene expression have been inhibited. More importantly, ethanol treated dramatically decreased it in the nucleus expression, however over-expressed miR-203, the nucleus localization of Lipin1 is increased.

## Conclusion

In summary, miR-203 is a new target in the AFL and negatively regulated Lipin1 in AML-12 cells. Importantly, miR-203 functioned as a new mediator and has therapeutic potential for AFL by regulation of lipid metabolism. More importantly, our study indicates the cross-talk of miR-203 with Lipin1 and may provide a new insight into double functions of Lipin1 in ethanol-induced liver lipid accumulation.

## Ethics Statement

Before the experiments, all procedures were approved by the ethical committee of the Anhui Medical University.

## Author Contributions

X-YC: conceived and designed the experiments. J-DL and X-YL: performed the experiments. XY, X-MM, and CH: analyzed the data. JL: contributed the reagents, materials, analysis tools and wrote the paper. All authors reviewed and approved this manuscript.

## Conflict of Interest Statement

The authors declare that the research was conducted in the absence of any commercial or financial relationships that could be construed as a potential conflict of interest.
